# Consumer Food Waste Behavior among Emerging Adults: Evidence from China

**DOI:** 10.3390/foods9070961

**Published:** 2020-07-21

**Authors:** Wang-Chin Tsai, Xuqi Chen, Chun Yang

**Affiliations:** 1 Department of Creative Design, National Yunlin University of Science &Technology, Yunlin 640, Taiwan; wangwang@yuntech.edu.tw; 2 Department of Agricultural and Resource Economics, University of Tennessee, Knoxville, TN 37996, USA; Xchen88@utk.edu; 3 Graduate School of Design, National Yunlin University of Science &Technology, Yunlin 640, Taiwan

**Keywords:** emerging adulthood, food waste, theory of planned behavior, environmental concerns, structural equation modeling

## Abstract

With the aggravation of global climate change, the issue of environmental protection has become the focus of global attention, and countries all over the world have devoted themselves to the sustainable development of resources to reduce the negative impact of the environment on human society. Reducing the resource waste is an important aspect of the sustainable development, among which food waste is a critical part. According to a report of the United Food and Agriculture Organization of the United Nations (FAO), 35% of food is wasted during consumption. Although households are the main contributors to food waste during consumption, the situation in university canteens cannot be ignored. As universities have a high degree of social influence, some policies and activities are piloted in universities, and then, promoted to society after achieving significant results. In future social development, the food waste behavior of consumers at the early stage of adulthood will have a significant impact on society. Therefore, it is necessary to understand the factors that lead to food waste by early adulthood consumers. This study focuses on food waste by end consumers and explores factors in the food waste behavior of the emerging adulthood consumer, which can be used as a reference for improving food waste in schools, governments, and other related industries in the future. The results show that the model of factors influencing the food waste behavior of emerging adulthood consumers established in this study is acceptable. According to the analysis results of the structural equation modeling (SEM), it can be seen that the influences of environmental concerns on the attitude toward behavior, subjective norms, and perceived behavioral control are ranked first, second, and third, respectively. While emerging adulthood consumers have a high degree of independence and self-awareness, schools, governments, media networks, and other related industries also need to establish a more complete system and form of cherishing food, in order to encourage emerging adulthood consumers to change their behavior and attitude spontaneously.

## 1. Introduction

### 1.1. Research Background and Motives

The climate anomaly is a global “tragedy of the commons” [1] that has had a dramatic impact on the environment. Unpredictable natural disasters have caused great social, economic, and human losses, and such incalculable losses have made countries around the world pay more attention to environmental issues. Large organizations, such as the United Nations, have launched relevant investigations and research in the hope of ameliorating the effects of an abnormal climate. In “Transforming our World: The 2030 Agenda for Sustainable Development”, proposed by the United Nations [2], item 12 is “ensuring sustainable consumption and production, making the international community actively develop towards green growth and circular economy”. It is pointed out that nearly 1/3 of the world’s food is wasted by people [3], and over 820 million people suffer from hunger, which means that one out of every nine people in the world does not have enough food to eat [4]. Food waste refers to the loss of edible food by people at the retail or sales end, which is usually caused by the fact that consumers discard edible food due to excessive purchasing [5], or that the appearance of food is not up to standard. The food is discarded by the manufacturer [6]. These are usually deliberate acts of waste [7]. When consuming large amounts of food, water, energy, and land investments in the production of such food are also consumed inefficiently [8]. People should reduce food loss and waste in production and consumption to avoid further sacrificing the earth’s biodiversity [9]. This is also an essential part of the sustainable development goals of the United Nations.

**Figure 1:**
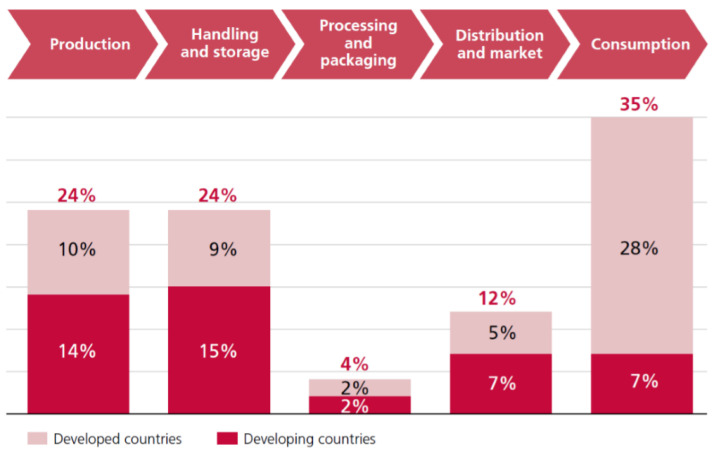
Stats of food loss in food supply chains (source: Gender and Food Loss in Sustainable Food Value Chains of Food and Agriculture Organizations [10]).

According to the report of the Food and Agriculture Organization (FAO) [10] in 2018 (Figure 1) 35% of food is wasted during consumption. Although households are the main contributors to food waste during consumption [11], the situation in university canteens cannot be ignored [12]. As universities have a high degree of social influence, some policies and activities are piloted in universities, and then, promoted to society after achieving significant results. Current studies on university food waste mainly focus on Africa [13], South America [14], and North America [15,16,17]. To date, most researchers in China have studied food waste from a macroscopic perspective, such as the definition of food waste, food deterioration [18], and food loss [19], as well as the causes of food waste and policy impacts [20], while there are few studies on students’ food waste in universities. Therefore, it is feasible to conduct a case study of China. As the most populous and fastest-growing country in the world, China’s per capita income continues to increase, and correspondingly, extravagant consumption has become a trend, which includes food waste. Although China has been promoting the Clean Plate Campaign since 2013, the results are still unsatisfactory, and food waste still exists, which constrains the sustainable development of China’s society and economy. In 2015, the total amount of food waste in China’s cities amounted to approximately 17 to 18 million tons, which was half of Hebei Province’s production in the same year (33.638 million tons) [21]. A large amount of food waste causes excessive carbon emissions (124 g CO_2_ eq. per person per day globally, 152 g CO_2_ eq. per person per day in China, and 315 g CO_2_ eq. in high-income areas) [22]. In addition, due to the lack of promoting garbage classification and recycling in China, urban areas are not sanitary. When kitchen waste is mixed with other garbage, it will produce a lot of harmful gases after incineration, which will affect people’s living environments. Such large amounts of food waste have affected people’s living environments, and the government has to pay extra costs to properly handle food waste, thereby causing losses to the economy and environment [7]. If we can reduce the production of food waste, the cost can be saved to give back to society and the environmental burden can be reduced. In future social development, the food waste behavior of consumers at the early stage of adulthood will have a significant impact on society. Therefore, it is necessary to understand the factors that lead to food waste by emerging adulthood consumers.

### 1.2. Research Purpose

China is part of a global effort to address the food crisis with a sustainable development plan focused on reducing food waste. However, according to the data provided by WWF and the Chinese Academy of Sciences, there is still a lot of food waste in China, resulting in serious problems [21]. This study explores the food waste behavior of consumers in early adulthood, discusses these consumption behaviors, analyzes the main factors that affect the food waste behaviors of early adulthood consumers, and examines the relationships between these factors. Then, improvement suggestions are offered for the reference of relevant organizations.

### 1.3. Research Scope

Food waste is mainly caused by food “production”, “storage and transportation”, “sales”, and “consumption” [8]. This study focuses on discussing food waste at the consumption end, which involves a series of behaviors, such as cooking, eating, and discarding, after consumers purchase food.

To ensure the concentration and effectiveness of the samples, the emerging adulthood consumers of this study are university students, and no questionnaires were given to other consumers of the same age.

## 2. Relevant Studies

### 2.1. Environmental Concerns and Environmental Education

#### 2.1.1. Environmental Concerns

Environmental awareness refers to people’s inner reactions [23], as well as the behavior and psychological state of environmental commitment [24,25]. Environmental awareness is also a kind of belief. While “belief” refers to a person’s descriptive idea of something, an attitude refers to a person’s consistent evaluation, feeling, and the tendency of something or view [26]. Attitude is usually regarded as a factor that can directly influence behavioral tendencies or behavior, while belief is often regarded as an important influencing factor or prerequisite factor [27,28]. In the study of consumer behavior, values affect consumer values, and thus, the product attribute belief. Consumer values will influence the product attribute belief, while the product attribute belief will influence product attitudes [29].

Environmental concerns refer to a person’s views of environmental issues, the degree of concern about environmental problems [30], or a strong attitude towards environmental protection [31]. The term is also used as one of the important predictors of environmental awareness. Consumers’ behavioral decisions often depend on their attitudes towards the environment [32]. When individuals have better environmental awareness, they may be more environmentally friendly than others.

#### 2.1.2. Environmental Education

In order to achieve sustainable development, environmental education usually focuses on the educational functions of the natural and ecological environment, and how humans manage their behaviors and ecosystems [33]. As universities often develop the knowledge, skills, and tools to create a sustainable future through education, research, policymaking, outreach, and activities [34]. In the last decade, universities have conducted environmental education around the world, as they try to achieve the ideal environment through social innovation at a small scale and extent it throughout society [35]. Green suggested that exploring sustainable ideas through educational courses can be key to influencing student attitudes and behavior [36]. Dagiliūtė and Liobikienė found that sustainable education courses could help develop students’ environmental protection consciousness and environmental knowledge [37], which are the main driving force of environmentally friendly behavior [38]. According to the above research, we can see that integrating university courses into sustainable development activities is one of the important ways to achieve sustainable education [39].

China got a late start in environmental education. In 1999, China carried out their eighth basic education curriculum reform, which formally included environmental education in the curriculum of primary and secondary schools, while only a few universities set up corresponding environmental education courses [40]. When environmental education courses conflict with other courses, environmental education courses are often ignored, resulting in a lack of awareness of environmental issues and understanding of relevant laws and regulations among students and the public [41].

### 2.2. Emerging Adulthood and Food Waste Behavior

Emerging adulthood is between adolescence and adulthood, with a focus on ages 18–25. Although young people at this stage are no longer teenagers, they are still different from adults in terms of cognition, self-definition, emotional control, and behavior [42]. There is a rather long transition period in emerging adulthood. Young people who are separated from their teenage years are unable to immediately assume the responsibilities of adults. Therefore, they need a period of self-exploration to develop their life process and career planning through multiple role tests and explorations [43,44].

According to the relevant literature, age is negatively correlated with food waste behavior, and the phenomenon of food waste is the most serious among young people [45,46]. Young people spend less time on cooking and prefer fast food, while older people have more cooking skills and more time to engage in cooking activities [47]. As young people often buy fast food, seldom cook food, and have no idea about food materials, they generally have a low awareness of food waste and mistakenly believe that they have not caused much waste [48]. Therefore, it is necessary to guide emerging adult people to establish correct consumption concepts and values in order to reduce food waste behaviors and phenomena.

### 2.3. Theory of Planned Behavior (TPB)

The Theory of Planned Behavior (TPB) is based on the Theory of Reasoned Action (TRA) [49]. According to Ajzen’s theory, personal behavior intention is influenced by three factors: (1) the attitude toward behavior, (2) the subject norm, and (3) perceived behavioral control [50,51]. TPB has been widely used in various fields of personal behavior, such as automobile and transportation [52,53,54], education [55,56,57], the environment [58,59], and medical treatment [60,61]. It is also often used to study consumer behavior in the food field [62,63,64,65,66].

In the TPB model, actual personal behavior is determined by personal behavior intention. Behavior intention determines the willingness of individuals to participate in specific behaviors [51]. The attitude in TPB refers to the attitude of an individual toward behavior. The subject norm refers to an individual’s psychological tendency and may be influenced by other factors, including social pressure [51,67,68]. Furthermore, perceived behavioral control refers to the difficulty of completing a specific behavior [67,68], and involves various factors, such as time, money, experience, and information.

**Figure 2:**
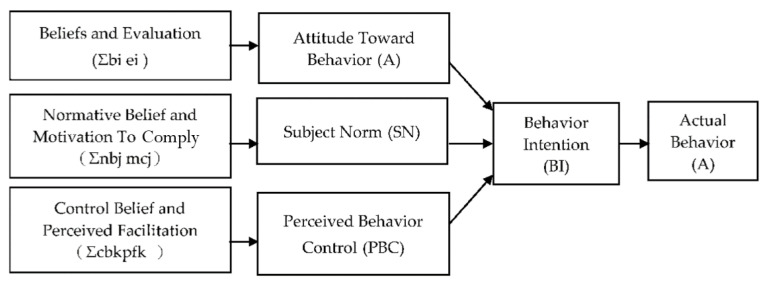
Theory of Planned Behavior (Source: [49]).

The attitude, subject norm, and perceived behavioral control also influence each other, and are constituted by three different beliefs: behavioral beliefs, normative beliefs, and control beliefs. Behavioral beliefs influence people’s attitudes toward behavior. People evaluate the results of behavior through behavioral beliefs and build attitudes toward behaviors. Subjective normative beliefs refer to the social pressure that people feel when engaging in behaviors, thus shaping people’s subjective normative framework. Control beliefs are the construction basis of perceived behavioral control. These three beliefs also have a profound impact on individual behavioral intentions [67]. TPB is shown in Figure 2.

## 3. Research Method and Hypothesis

### 3.1. Research Structure

**Figure 3:**
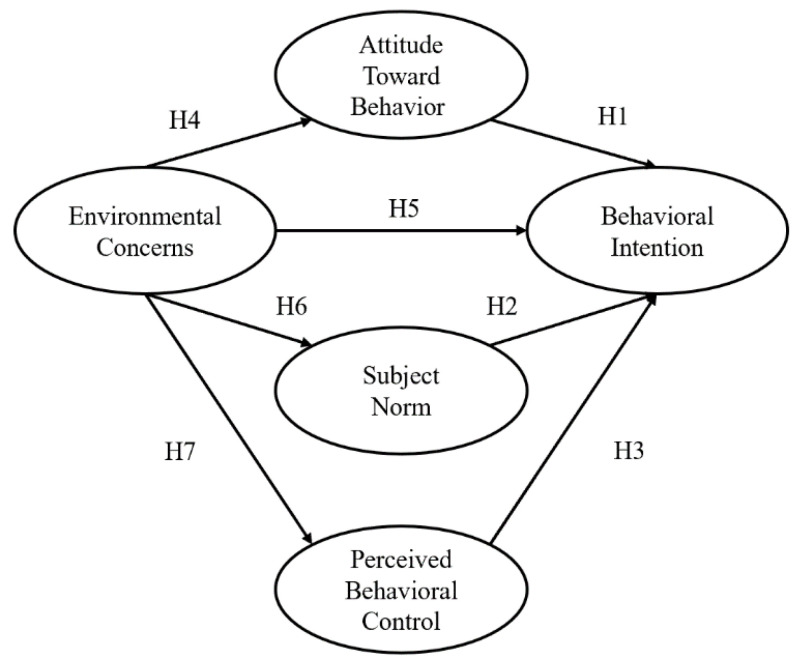
Research structure.

The issue of food waste has attracted an increasing amount of global attention, and people’s environmental awareness has gradually increased [69]. According to previous studies, environmental concerns (ECs) are associated with the three aspects of TPB and can affect people’s behavioral intentions; therefore, adding variables regarding environmental awareness to the TPB model is conducive to improving its reliability and effectiveness [70,71,72,73,74,75,76,77,78]. Ajzen suggested that the TPB model can be applied to food consumption decisions [79]. Therefore, this study used TPB to predict the food waste behavior of emerging adulthood consumers, understand the basis of their beliefs, explore the causes of their behavioral intention, and then develop promotional activities to reduce food waste behavior. At the same time, ECs were added as the premise in the TPB model [51,67,71,78], as shown in Figure 3.

### 3.2. Research Process and Method

This study is divided into three stages. The first stage clarifies the context of the food waste issue through the literature, defines the subject and scope of this study, and establishes the research model regarding the research issue.

The second stage conducts a questionnaire survey. Samples are collected through web-based questionnaires to understand the factors that lead to food waste by early adulthood consumers. The reliability of the questionnaire is investigated through pre-testing, and after passing reliability and validity testing, the formal questionnaires are distributed.

In the third stage, the formal questionnaires are recovered, and structural equation modeling (SEM) is adopted to analyze the factors of the food waste behaviors of early adulthood consumers, explore the relationship between them, form the final research model, and discuss its implications.

### 3.3. Research Hypothesis

Based on the previous discussion, this study proposes several hypotheses about the factors that influence the food waste behavior of emerging adulthood consumers:

**Hypothesis 1. (H1)**: There is a significant positive correlation between attitude and the emerging adulthood consumer’s behavioral intention towards food waste behavior.

**Hypothesis 2. (H2)**: There is a significant positive correlation between the subjective norm and the emerging adulthood consumer’s behavioral intention towards food waste behavior.

**Hypothesis 3. (H3)**: There is a significant positive correlation between perceived behavioral control and the emerging adulthood consumer’s behavioral intention towards food waste behavior.

**Hypothesis 4. (H4)**: There is a significant positive correlation between environmental concerns and the attitude of the emerging adulthood consumer toward food waste behavior.

**Hypothesis 5. (H5)**: There is a significant positive correlation between environmental concerns and the behavioral intention of the emerging adulthood consumer toward food waste behavior.

**Hypothesis 6. (H6)**: There is a significant positive correlation between environmental concerns and the subjective norm of the emerging adulthood consumer toward food waste behavior.

**Hypothesis 7. (H7)**: There is a significant positive correlation between environmental concerns and the perceived behavioral control of the emerging adulthood consumer toward food waste behavior.

### 3.4. Definition and Measure of the Variables

In this study, the theoretical framework of the factors that influence emerging adulthood consumers’ food waste behavior was divided into five aspects: attitude, subject norm, perceived behavioral control, behavioral intention, and actual behavior. The items of the questionnaire were designed according to the research topic and the related literature, as shown in Table 1.

## 4. Research Results and Discussion

### 4.1. Sample Selection

As a major province of education in China, Jiangsu has attracted a large number of students from all over the country. According to the statistics of the Ministry of Education of the PRC in 2018 [80], Jiangsu has 77 undergraduate universities, accounting for the largest number of undergraduate institutions in China. The total number of undergraduate students in Jiangsu is 1,121,239, ranking third in China. Therefore, this study chose the universities of Jiangsu as the sample to understand the factors of food waste behavior among emerging Chinese adulthood consumers. Ethical approval for this study was obtained from the National Cheng Kung University Human Research Ethics Committee.

### 4.2. Descriptive Analysis of Demographic Variables

In terms of the distribution of the questionnaire sample in this study, data were collected through an online survey completed by 400 university students from 48 universities according to the proportion 3 (Southern Jiangsu): 1 (Northern Jiangsu) of the universities in Jiangsu Province. The questionnaire is divided into two parts. The first part is consisted of single-choice questions and we investigated the respondents’ gender, grade and thoughts and conditions about food waste. The questions of the second part are in the form of Likert’s seven-point scale, from 1 (strongly disagree) to 7 (strongly agree) (see Table 1). From September to October 2019, a total of 400 questionnaires were distributed, and after eliminating invalid samples (samples with logic errors or too many same options), 368 valid questionnaires were recovered, for a recovery rate of 92%. The questionnaire data were analyzed by structural equation modeling (SEM). Generally speaking, the number of samples required by SEM should be between 200 and 500 [81]. If the number of samples exceeds 500, the chi-square value will be greatly inflated when the maximum likelihood method is applied, resulting in poor model matching. Jackson suggested that the ratio (*p*: *n*) of the estimated parameter to the sample size should be 1:20, as based on the maximum likelihood method, while 1:10 is the minimum requirement of the sample. If the ratio is less than 1:5, it lacks reliability. Therefore, the estimated parameters of the questionnaire in this study were 18, the sample number was 368, and the ratio was 1:20.44, which is higher than the ratio of 1:20, as required by Jackson [82]. Therefore, we considered that the sample size of the questionnaire in this study was reasonable, and subsequent statistical analysis was carried out accordingly. According to the data of the subjects in the valid questionnaires, statistical analysis was carried out to understand the gender and grade distribution of the sample. The distribution of population variables is shown in Table 2.

In addition, this study collected the current situations of the dining and food waste of the respondents (survey samples). Table 2 shows that most of the respondents in this study chose university canteens (265, 72.01%) and take-out (224, 60.87%), while only a small number chose to eat in a restaurant (58, 15.76%). Most of the respondents had little (214, 58.15%) or nearly no leftovers (88, 23.91%), and only about 18% of the respondents produced a lot of leftovers (66, 17.94%). Regarding the disposal of leftover food, 323 people (87.78%) chose to throw it in the trash, 55 (14.95%) chose to keep it for their next meal, 41 (11.14%) chose to use leftovers as food for pets or stray animals, and 59 (16.03%) chose other. In addition, 258 people (70.1%) felt guilty when they threw away food, 81 people (22.01%) only felt a little guilty, 14 people hardly minded (3.8%), and 15 (4.08%) did not mind at all. According to the above descriptions, it can be seen that most of the respondents are concerned about leftovers and cherishing food, will try their best to finish their meals, and feel sorry about leftovers.

### 4.3. Convergent Validity and Discriminant Validity

This study conducted a reliability test on the survey data. The reliability test was used to measure the reliability and consistency of the questionnaire data. The results showed that all of the questions in this study were highly reliable and valid (Cronbach’s α coefficients of all variables were greater than the standard value of 0.6), so the formal questionnaires were distributed (see Table 3).

### 4.4. Convergent Validity and Discriminant Validity

This study used Confirmatory Factor Analysis (CFA) and Maximum Likelihood Estimation (MLE) to measure the reliability and validity, path coefficient, convergent validity, and discriminatory validity of the questionnaire data [83]. Table 4 shows the relevant criteria of the structural equation model [84].

In Table 4, each standard factor load is between 0.479 and 0.947, the composite reliability of the research dimensions is between 0.72 and 0.945 (>0.7) [85], and Average Variance Extracted (AVE) is from 0.478 to 0.811 (>0.5) [84,86]. This shows that the data of this study have a considerable degree of reliability, validity, internal consistency, and aggregation.

The results of Fornell and Larcker [84] were used to test the discriminant validity of this study. If the AVE square root of each dimension is higher than the correlation coefficient between dimensions, it means that the model has discriminant validity.

As shown in Table 5, the AVE square root of each dimension in the diagonal line is higher than the correlation coefficient beyond the diagonal line; hence, each dimension has a high level of discriminant validity.

### 4.5. Structural Model Fit Text

SEM measures the non-observable index according to observable indices [81,87]. The attitude, environmental concerns, subject norm, perceived behavioral control, and behavioral intention were measured according to the previous research hypothesis and model. After the data were input into the model, the degree of fitness of the model was evaluated by using a variety of evaluation indices. The evaluation result is shown in Table 6, which shows that most indices meet the criteria, indicating that the model of this study exhibits a good fitness.

**Figure 4:**
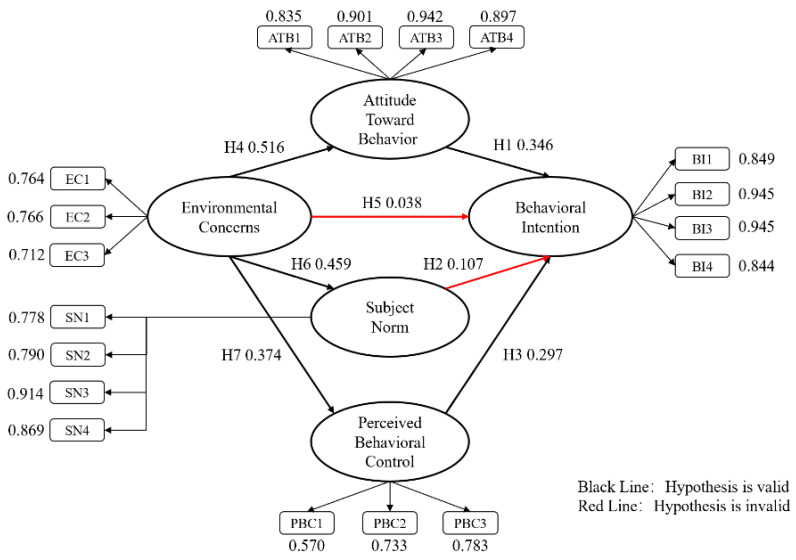
Research structure pattern diagram.

As shown in Table 7, the AMOS model hypothesis test results show that all test results are consistent with the hypotheses except H2 and H5, indicating that attitude toward behavior (ATB) and perceived behavioral control (PBC) have a significant impact on behavioral intention (BI), while ECs have a significant impact on ATB, subjective norm (SN), and PBC. The specific visualization is shown in Figure 4.

### 4.6. Discussion

The purpose of this study is to establish a theoretical framework of factors influencing the food waste behavior of emerging adulthood consumers, the structural equation model is used to determine the key factors influencing behavior intention, and the conclusions and suggestions are put forward as a reference for future research and related food industry and enterprises. This study also hopes to put forward some consideration factors for the solution of food waste problems by emerging adulthood consumers in universities. Based on the above hypothesis results, the following discussions were carried out in this study.

H1 is valid. There is a significant positive correlation between attitude and the emerging adulthood consumer’s behavioral intention towards food waste behavior, which shows that the attitude towards behavior in this study influences the behavioral intention. The direct impact of attitudes on intentions has been demonstrated in TRA [88], TPB [49,51], and TAM [89], which demonstrates that attitude is a favorable predictor of the behavioral intention of food waste in early adulthood consumers. It can be seen from the items of attitude towards behavior that emerging adulthood consumers think that reducing food waste has a positive impact on environmental protection. Additionally, reducing food waste helps improve the quality of life, which is a smart promotion-worthy approach. Some consumers feel guilty when they or other people waste food, so they will reduce food waste. The remaining consumers think that food waste has no direct impact on others and choose to ignore it [48,90]. Therefore, the more positive the attitude towards behavior is, the more consumers have the tendency of planning shopping and not wasting food, and the more likely such consumers are to become supervisors to prevent others from wasting food.

H2 is invalid. There is no significant positive correlation between the subjective norm and the emerging adulthood consumer’s behavioral intention towards food waste behavior, indicating that the subjective norm in this study does not influence the behavioral intention. It can be seen from the items of the subjective norm that important people (family members, friends, and colleagues) have a small effect on emerging adulthood consumers, and education, media, and Internet information also have a small effect on them. The reason for this may be that young people who have just become independent and are studying or working away from home have freed themselves from the constraints of their families and elders, and reduced their self-discipline [91]. In addition, schools, governments, and media have not given enough publicity to reducing food waste [92], which results in emerging adulthood consumers thinking that schools, the government, and the media have no direct impact on whether they need to cherish food.

H3 is valid. There is a significant positive correlation between perceived behavior control and the emerging adulthood consumer’s behavioral intention towards food waste behavior, indicating that the attitude towards behavior in this study has an impact on the behavioral intentions. It can be seen from the items of perceived behavioral control that the consideration of the environment and self-status is one of the main influencing factors of food waste behavior for emerging adulthood consumers. If they have a sufficient understanding of food waste, they will reduce their food waste behaviors [18], and they will try to eat their food, even if it is not palatable. In addition, when they have meals with family members, friends, or colleagues, they will increase their self-restraint to reduce food waste [91]. Such passive behaviors of cherishing food can enhance others’ impressions of them [93].

H4 is valid. There is a significant positive correlation between environmental concerns and the attitude of the emerging adulthood consumer toward food waste behavior. The results show that environmental concerns in this study have an impact on the attitude towards behaviors, which indicates that when emerging adulthood consumers pay greater attention to environmental problems, it will help them reduce their food waste [94]. Many people are aware of the human damage to the environment, as well as the coming environmental crisis and resource shortage. Therefore, they will identify more with the attitude and practice of cherishing food, which will influence their behaviors.

H5 is invalid. There is no significant positive correlation between environmental concerns and the behavioral intention of the emerging adulthood consumer toward food waste behavior. This shows that the environmental concerns in this study have no influence on the behavioral intention, indicating that environmental concerns cannot directly affect the behavioral intention of food waste behavior of emerging adulthood consumers, but should be converted into information to transform the behavioral intention through changing the consumers’ self-consciousness and the interference of external factors.

H6 is valid. There is a significant positive correlation between environmental concerns and the subjective norm of the emerging adulthood consumer toward food waste behavior, which shows that environmental concerns in this study have an impact on the attitude towards behaviors. This indicates that drawing attention to environmental issues will affect the important people (family, friends, and colleagues) of emerging adulthood consumers’ environmental protection concepts, and will have a specific impact on emerging adulthood consumers’ own concepts and thoughts. The greater the attention paid to environmental issues, the greater the influence of important people (family, friends, and colleagues) on emerging adulthood consumers.

H7 is valid. There is a significant positive correlation between environmental concerns and the perceived behavioral control of the emerging adulthood consumer toward food waste behavior, which shows that environmental concerns in this study have an impact on perceived behavioral control. For emerging adulthood consumers, environmental concerns can become one of their external considerations for food waste. Emerging adulthood consumers who pay more attention to environmental issues will know more about food waste, and thus have more correct cognition, which will influence their behavioral intentions.

Overall, this study has developed the TPB model as the theoretical framework, combined with environmental concerns, and produced a structural equation model (SEM) to study the causes of the behavioral intention of food waste in early adulthood consumers. Structural equation modeling has been proven to be suitable for the research of food waste [95]. Combining the TPB model with environmental concerns is an innovation of this study. With the exception of χ^2^, all model fit indicators exceed the recommended levels. Therefore, the influencing factor model of the food waste behavior of emerging adulthood consumers, as established in this study, is acceptable, indicating that this model has some effect on explaining the food waste behavioral intention of emerging adulthood consumers. In this study, attitude and perceived behavioral control have a significant impact on behavioral intention, while the subject norm shows no obvious effect, which is consistent with the findings of Mondéjar-Jiménez et al. regarding food waste [96]. Environmental concerns fail to have any significant impact on behavioral intention, and instead, influence it through attitude and perceived behavioral control, which means that environmental concerns have an indirect effect on the behavioral intention [97], and represents that self-awareness in early adulthood consumers is an important determinant of food waste. Self-awareness consists of attitudes, thoughts, and emotions. When consumers become aware of food waste and realize that reducing food waste will benefit the environment through their own perceptions of environmental concerns, or when consumers develop corresponding environmental concerns [98], they will reduce their relevant food waste intentions and behaviors [99]. In contrast, the subject norm has no significant effect on the behavioral intention, which in turn restricts the ability of environmental concerns to affect the behavioral intention through the subject norm. This suggests that environmental concerns, such as those of early adulthood consumers, can impact different groups of people (families, friends, and peers, including the consumers in early adulthood themselves). However, in contrast to the results of perceived behavioral control and the subject norm, early adulthood consumers have a higher self-awareness and prefer to make decisions on their own. Therefore, they are less likely to accept advice from and be influenced by those around them, even those who are important to them. Therefore, to reduce the food waste behaviors of early adulthood consumers, efforts should start with changing and influencing their behaviors through education on food appreciation, in order to induce their own internal changes.

## 5. Conclusions and Suggestions

### 5.1. Conclusions

Based on TPB, environmental concerns are regarded as the source of attitudes toward behavior, subject norm, perceived behavioral control, and behavioral intention in this study. The purpose of the study was to establish a theoretical framework for influencing the food waste behavior of emerging adulthood consumers, and then use the structural equation model to determine the main factors affecting the behavioral intention. Through an analysis of the relevant effects, we have shown that most of the aspects have a direct or indirect impact on the food waste behavior of emerging adulthood consumers.

Overall, five of the seven hypotheses are valid. Among them, environmental concerns rank first, second, and third in terms of the influence of the attitude toward behaviors, subjective norms, and perceived behavioral control, respectively, indicating that environmental concerns must receive greater attention by emerging adulthood consumers, as well as the people around them, in order to have a considerable influence on them. Environmental concerns affect the behavioral intention through the attitude toward behavior and perceived behavioral control. Although environmental concerns have a high impact on subject norms, they will not affect the behavioral intention, which means that emerging adulthood consumers have a high degree of independence and self-awareness. They will determine their own ideas, attitudes, and behavioral intentions regarding food waste behaviors according to their own experience and cognition, and be less affected by other people, schools, or network information [100]. However, this does not mean that external factors have no significant impact on emerging adulthood consumers; on the contrary, we believe that the current impact insufficient. There are many factors causing food waste in schools, including the food surplus [13,101], canteen atmosphere [102], and school system [103], which make emerging adulthood consumers in this environment imperceptibly accustomed to the behavior of food waste. Schools need to do more to influence emerging adulthood consumers to change their food waste behavior, and the government should establish a national food education network to reduce food waste and excessive packaging while ensuring national health and safety and releasing resources for other areas in need. Meanwhile, consumers’ behavioral attitudes can be influenced by media networks and online celebrities. Through the above points, emerging adulthood consumers can be guided to produce or strengthen their concept of cherishing food, thus changing their behavioral attitudes spontaneously.

### 5.2. Research Limitations and Future Research Suggestions

Some limitations of this study may indicate future research directions, as follows:

1. This study explored the determinants of the food waste behavior of consumers at the early stage of adulthood (college students), but did not discuss young adults from higher education institutes or other consumers. Future researchers could explore the related factors of food waste among different groups.
2. This study established a research model based on the theory of planned behavior and environmental concerns. However, the explanatory power of the model is still inadequate, and there may be other unknown dimensions that are not discussed in this study. In the future, researchers can introduce different theories for research. For the follow-up study, we could thoroughly analyze the internal influence factors, such as the emotions and thoughts of early adulthood consumers, and add new dimensions based on this study, including second-order dimensions and intermediary variables, to improve the explanatory power of the model and perfect it;
3. Due to the limitations of time and resources, this study only collected questionnaires from Jiangsu, China. Consumers in other regions may have different views on the subject of this study due to the differences between China’s regions. In the future, researchers could explore the situations in different regions and provide a reference for governments, schools, and related enterprises.
4. Finally, we think that with direct questioning, social desirability bias may influence the answers of participants. Subsequent researchers may be able to use interviews and other methods to specifically verify the true ideas of consumers.

## Figures and Tables

**Table 1:** Definition of variable operability and reference scales.

Research Variable	Operability Definition	Code	Questions	Reference Scale
Environmental concerns	Environmental concerns refer to the individual’s views or concerns about environmental issues, which may affect the individual’s attitude or behavior.	EC1	Humans are seriously abusing the environment, and the garbage problem is getting worse	[71,78]
		EC2	For the sake of their own future, humans have to live in harmony with nature	
		EC3	I’m worried about the global environment condition and how it may impact my future	
Attitude toward behavior	The attitude towards behavior is the attitude used to measure the positive and negative results of food waste behavior; that is, the actual attitude and evaluation of the food waste behavior of emerging adulthood consumers.	ATB1	I believe reducing food waste will have a positive effect on environment protection	[51,67,68]
		ATB2	I think reducing food waste is helpful to improve the quality of life	
		ATB3	I believe it is a wise move to reduce food waste	
		ATB4	I’m willing to reduce the damages to the environment through my own actions	
Subject norm	The subject norm refers to the degree to which the significant reference objects (individuals or groups) of emerging adulthood consumers regulate them.	SN1	For me, the opinions of families, friends, and peers on food waste are important	[51,67,68]
		SN2	I’ll change my behavior by following the opinions on food waste of families, friends, and peers that have influence over me	
		SN3	For me, the opinions of mass media, government policy, online information, experts, and salesmen on food waste are important	
		SN4	I’ll change my behavior by following the opinions on food waste of mass media, government policy, online information, experts, and salesmen that have influence over me	
Perceived behavioral control	Perceived behavioral control is used to measure the degree of behavioral control of subjects’ food waste behavior in most situations; that is, the degree of behavior execution under subjective judgment.	PBC1	Whether to waste food fully depends on me	[51,67,68]
		PBC2	For me, I won’t leave food, even if I don’t like it	
		PBC3	When I’m having meals with family, friends, and peers that have influence over me, they may stop me from wasting food	
Behavioral intention	Behavioral intention refers to the possibility of food waste by subjects in the future. In this study, the time span of behavioral intention is one month.	BI1	For environmental reasons, I have a strong desire to reduce food waste	[51,67,68]
		BI2	I’ll reduce my food waste in the following month	
		BI3	Reducing food waste delights me	
		BI4	I’ll spread the word to others to reduce food waste	

**Table 2:** Table showing basic sample data.

Sample	Category	Number	Percentage
Gender	Male	179	48.64%
	Female	189	51.36%
Grade	Freshman	43	11.68%
	Sophomore	106	28.8%
	Junior	132	35.87%
	Senior	87	23.64%
Location (multiple choice)	Cafeteria	265	72.01%
	Take-out	224	60.87%
	Restaurant	58	15.76%
Is there leftover food?	Quite a lot	66	17.94%
	Few	214	58.15%
	Nearly no leftovers	88	23.91%
How do you deal with leftover food? (multiple choices)	Keep it for the next meal	55	14.95%
	Discard as trash	323	87.78%
	Feed it to pets or stray animals	41	11.14%
	Others	59	16.03%
Do you feel guilty when you throw away the food?	Yes	258	70.1%
	A little guilty	81	22.01%
	Hardly minded	14	3.8%
	Did not mind at all	15	4.08%

**Data source**: Compiled by this study.

**Table 3:** Results of the questionnaires.

Dimension	Question	Cronbach’s α	Correlation Coefficient with the Total Scale Score	The *p*-Value in *t*-Test on an Independent Sample
Environmental concerns (ECs) Cronbach’s α = 0.803	EC1	0.747	0.635	0.000
	EC2	0.724	0.657	0.000
	EC3	0.723	0.658	0.000
Attitude toward behavior (ATB) Cronbach’s α = 0.940	ATB1	0.936	0.813	0.000
	ATB2	0.915	0.876	0.000
	ATB3	0.911	0.895	0.000
	ATB4	0.924	0.849	0.000
Subjective norm (SN) Cronbach’s α = 0.902	SN1	0.890	0.736	0.000
	SN2	0.886	0.756	0.000
	SN3	0.852	0.841	0.000
	SN4	0.867	0.803	0.000
Perceived behavioral control (PBC) Cronbach’s α = 0.740	PBC1	0.710	0.528	0.000
	PBC2	0.569	0.636	0.001
	PBC3	0.681	0.545	0.000
Behavioral intention (BI) Cronbach’s α = 0.944	BI1	0.937	0.833	0.000
	BI2	0.916	0.900	0.000
	BI3	0.915	0.902	0.000
	BI4	0.938	0.831	0.000

**Table 4:** Results for the measurement model.

Construct	Item	Significance of Estimated Parameters				Item Reliability		Construct Reliability	Convergence Validity
		Unstd.	S.E.	Unstd./S.E.	*p*-Value	Std.	SMC	CR	AVE
EC	EC1	1.000				0.761	0.579	0.803	0.576
	EC2	1.063	0.088	12.098	0.000	0.779	0.607		
	EC3	1.018	0.083	12.283	0.000	0.736	0.542		
ATB	ATB1	1.000				0.835	0.697	0.941	0.800
	ATB2	1.093	0.048	22.893	0.000	0.901	0.812		
	ATB3	1.047	0.044	23.956	0.000	0.941	0.885		
	ATB4	1.083	0.049	22.224	0.000	0.898	0.806		
SN	SN1	1.000				0.782	0.612	0.905	0.705
	SN2	1.141	0.070	16.295	0.000	0.789	0.623		
	SN3	1.184	0.061	19.375	0.000	0.912	0.832		
	SN4	1.099	0.061	18.132	0.000	0.869	0.755		
PBC	PBC1	1.000				0.479	0.229	0.720	0.478
	PBC2	1.191	0.146	8.148	0.000	0.634	0.402		
	PBC3	1.542	0.246	6.274	0.000	0.896	0.803		
BI	BI1	1.000				0.854	0.729	0.945	0.811
	BI2	1.082	0.041	26.544	0.000	0.947	0.897		
	BI3	1.092	0.041	26.343	0.000	0.947	0.897		
	BI4	1.016	0.047	21.531	0.000	0.849	0.721		

**Table 5:** Discriminant validity for the measurement model.

	AVE	EC	ATB	SN	PBC	BI
**EC**	0.576	**0.759**				
**ATB**	0.800	0.516	**0.894**			
**SN**	0.705	0.459	0.237	**0.84**		
**PBC**	0.478	0.374	0.193	0.172	**0.691**	
**BI**	0.811	0.376	0.448	0.257	0.396	**0.901**

**Table 6:** Model fit.

Model Fit	Criteria	Model Fit of the Research
MLχ^2^	The small the better	401.140
DF	The large the better	128.000
Normed Chi-sqr (χ^2^/DF)	1 < χ^2^/DF < 3	3.134
RMSEA	<0.08	0.076
SRMR	<0.08	0.086
TLI (NNFI)	>0.9	0.934
CFI	>0.9	0.944
GFI	>0.9	0.921
AGFI	>0.9	0.905

**Table 7:** Results of the SEM and hypothesis testing.

DV	IV	Unstd.	S.E.	Unstd./S.E.	*p*-Value	Std.	R^2^	Hypothesis	Text Results
ATB	EC	0.581	0.070	8.313	0.000	0.516	0.266	H4	Yes
SN	EC	0.522	0.072	7.213	0.000	0.459	0.211	H6	Yes
PBC	EC	0.335	0.064	5.256	0.000	0.374	0.140	H7	Yes
BI	EC	0.043	0.079	0.542	0.588	0.038	0.314	H5	No
	ATB	0.347	0.063	5.520	0.000	0.346		H1	Yes
	SN	0.106	0.057	1.865	0.062	0.107		H2	No
	PBC	0.375	0.108	3.462	0.001	0.297		H3	Yes
